# 3D reconstruction of coronary artery bifurcations from intravascular ultrasound and angiography

**DOI:** 10.1038/s41598-023-40257-8

**Published:** 2023-08-10

**Authors:** Wei Wu, Usama M. Oguz, Akshat Banga, Shijia Zhao, Anjani Kumar Thota, Vinay Kumar Gadamidi, Charu Hasini Vasa, Khaled M. Harmouch, Abdallah Naser, Xiarepati Tieliwaerdi, Yiannis S. Chatzizisis

**Affiliations:** 1 Center for Digital Cardiovascular Innovations, Division of Cardiovascular Medicine, Miller School of Medicine, University of Miami, Miami, FL USA; 2 Division of Cardiovascular Medicine, Leonard M. Miller School of Medicine, University of Miami Health System, University of Miami, 1120 NW 14th Street, Suite 1124, Miami, FL 33136 USA

**Keywords:** Cardiology, Interventional cardiology, Translational research

## Abstract

Coronary bifurcation lesions represent a challenging anatomical subset, and the understanding of their 3D anatomy and plaque composition appears to play a key role in devising the optimal stenting strategy. This study proposes a new approach for the 3D reconstruction of coronary bifurcations and plaque materials by combining intravascular ultrasound (IVUS) and angiography. Three patient-specific silicone bifurcation models were 3D reconstructed and compared to micro-computed tomography (µCT) as the gold standard to test the accuracy and reproducibility of the proposed methodology. The clinical feasibility of the method was investigated in three diseased patient-specific bifurcations of varying anatomical complexity. The IVUS-based 3D reconstructed bifurcation models showed high agreement with the µCT reference models, with r^2^ values ranging from 0.88 to 0.99. The methodology successfully 3D reconstructed all the patient bifurcations, including plaque materials, in less than 60 min. Our proposed method is a simple, time-efficient, and user-friendly tool for accurate 3D reconstruction of coronary artery bifurcations. It can provide valuable information about bifurcation anatomy and plaque burden in the clinical setting, assisting in bifurcation stent planning and education.

## Introduction

Interventional cardiology faces challenges in treating coronary bifurcation lesions because of low procedural success rates and increased rates of adverse cardiovascular events^1,2^. Because of these unique anatomical locations, different stenting strategies have been developed, which have been the subject of ongoing debate. To determine the best bifurcation stenting strategy and achieve favorable clinical outcomes, several factors, including bifurcation anatomy and disease extent, must be considered^3,4^. In recent years, interventional cardiologists have expressed the need for the three-dimensional (3D) representation of bifurcation anatomy and disease burden, as this can lead to a better understanding of the anatomical complexity of bifurcation disease and the optimization of stenting strategies.

Intravascular ultrasound (IVUS) is a powerful intravascular imaging technology that allows for the cross-sectional imaging of the coronary arteries. IVUS images have several anatomical features, including the lumen, external elastic membrane (EEM), and plaque materials. Several studies have proposed methods for combining IVUS and angiography images for 3D reconstruction of coronary arteries^5–23^; however, these approaches were focused on non-bifurcated vessels. Some studies have performed 3D reconstruction of bifurcations but relied on computed tomography (CT) images to correct the IVUS frame orientation, limiting their clinical applicability^24,25^. Furthermore, efforts to 3D reconstruct the plaque materials by IVUS have been limited to single vessels^21–23,26,27^.

In this study, we present a new method that combines IVUS imaging and angiography with advanced modeling techniques to perform 3D reconstruction of coronary bifurcations, including plaque materials. The study aimed to: (1) provide a detailed methodology for the 3D reconstruction of coronary bifurcations along with plaque materials, and (2) evaluate the methodology's accuracy, feasibility, and reproducibility in both patient-specific silicone bifurcation models and patient cases with varying degrees of disease.

## Methodology

All methods were carried out in accordance with the relevant guidelines and regulations. The angiograms and IVUS data used in the study were obtained from the Kyushu Medical Center, Fukuoka, Japan. The study was reviewed and approved by ethics committee of the National Hospital Organization Kyushu Medical Center (protocol number 20C035). Informed consent was obtained from all participants.

### Experimental studies

#### Experimental coronary artery models, flow chamber studies, and imaging procedures

**Figure 1:**
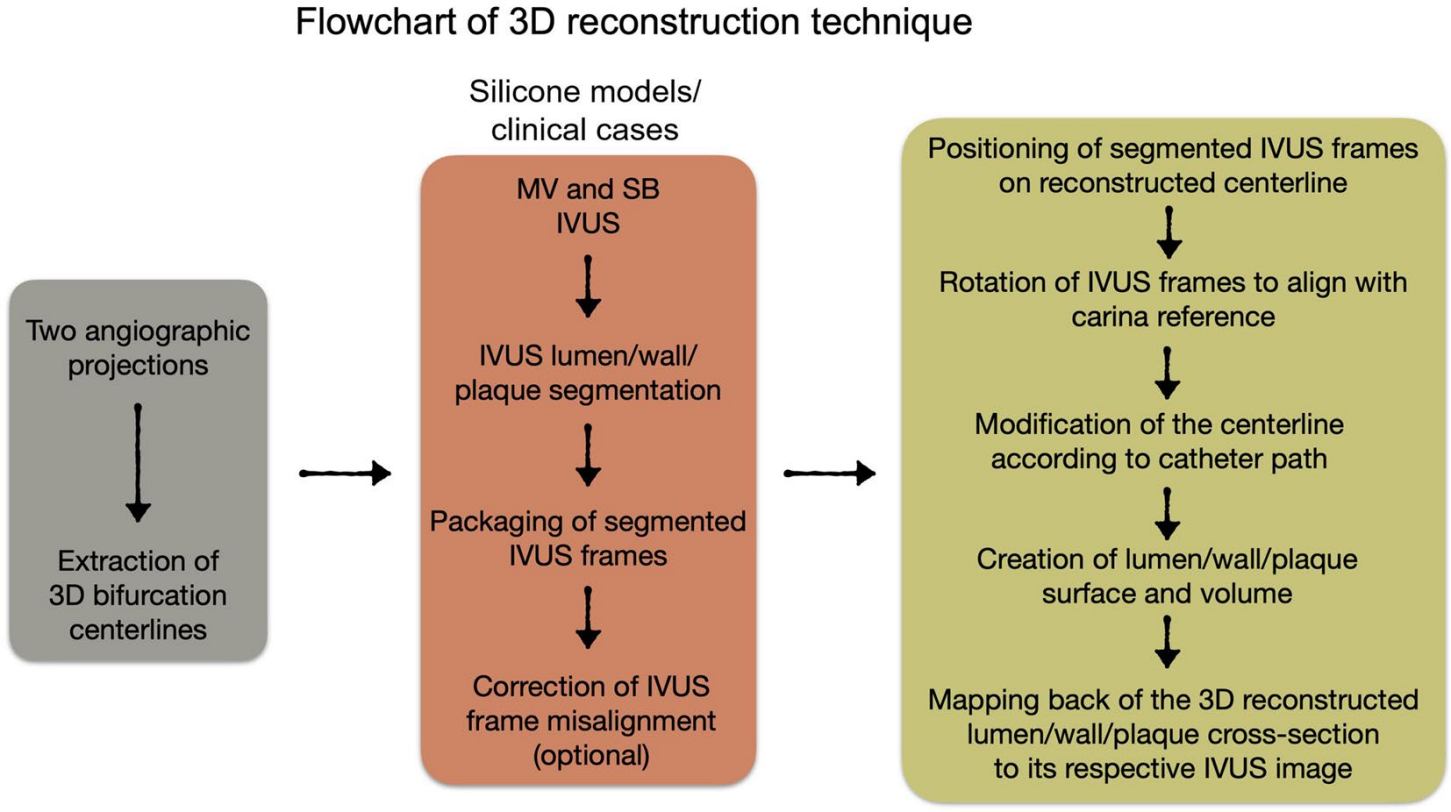
Flowchart of the 3D reconstruction of coronary artery bifurcation for patient-specific silicone models and clinical cases. IVUS, intravascular ultrasound; MV, main vessel; SB, side branch.

Figure 1 depicts a flowchart that explains the 3D reconstruction process. Three patient-specific silicone coronary bifurcation models were created using the previously described technique^28^. In brief, the 3D bifurcation geometries were created using two angiographic projections in 3D CAAS Workstation 8.2 (Pie medical imaging, Maastricht, The Netherlands). Negative moulds were created based on the geometries and 3D printed in acrylonitrile butadiene styrene. Polydimethylsiloxane was mixed with its curing agent before being poured into dry-clean moulds for curing. The silicone models were transferred to an acetone beaker to dissolve the acrylonitrile butadiene styrene material. The silicone models were placed in a custom-made flow chamber. A bioreactor circuit was connected to the flow chamber’s inlet and outlet, allowing steady blood-mimicking flow at a 100 ml/min rate at room temperature.

**Figure 2:**
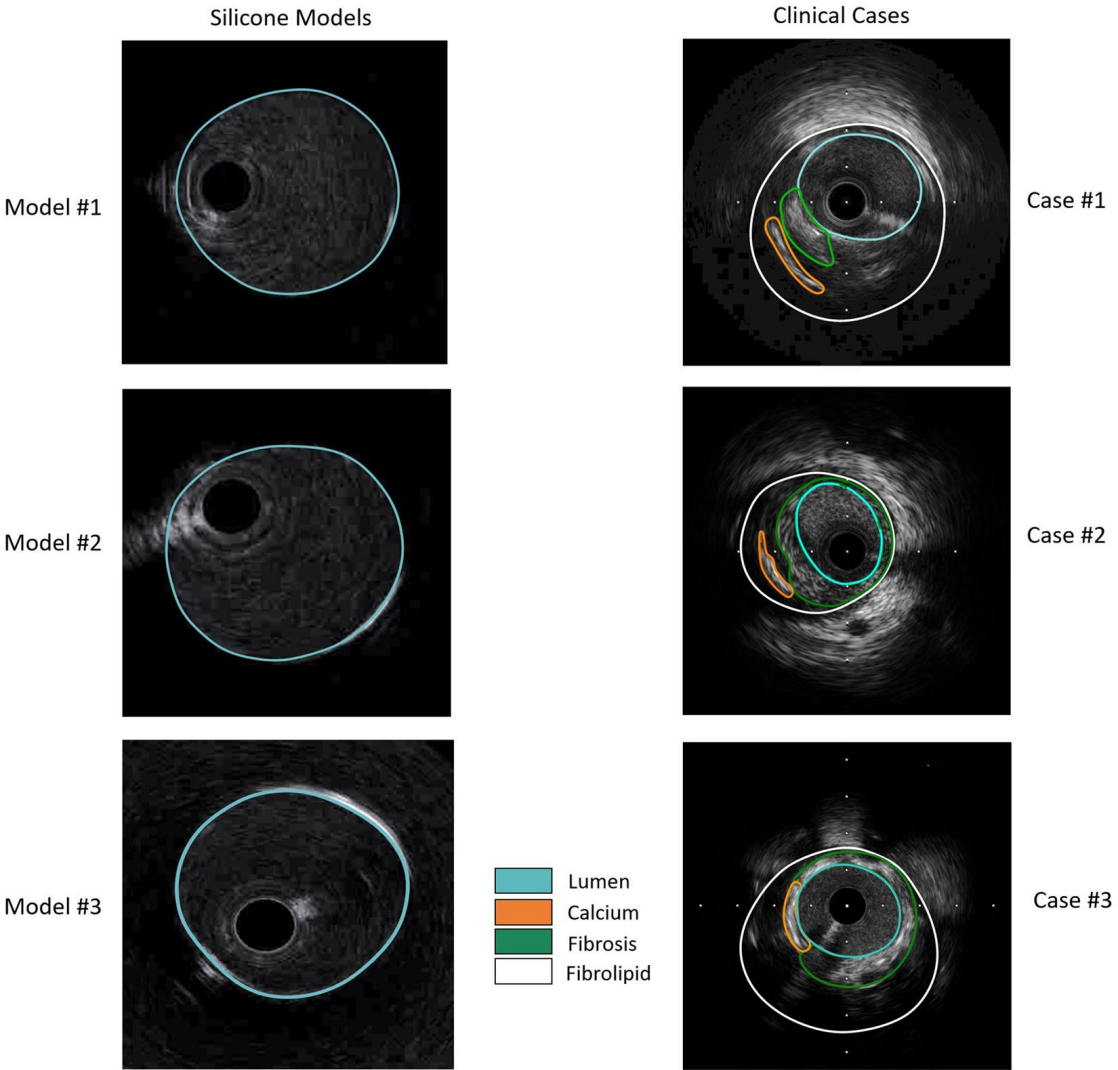
IVUS image segmentation of silicone models (lumen) and clinical cases (lumen, calcium, fibrolipid, and fibrosis).

The models were first imaged with angiography in two projections with at least a 30-degree difference in viewing angles to provide the main vessel (MV) and side branch (SB) centerlines. The three silicone models were subjected to IVUS imaging with Opticross 6 HD, 60 MHz (Boston Scientific, Marlborough, MA, USA). The imaging catheter was advanced using a 6F guiding catheter. An automatic pullback was performed at a constant speed of 0.5 mm/s or 1.0 mm/s, with a distance of 0.017/0.033 mm between two consecutive frames. All the pullback frames were analyzed offline, and the lumen sections were segmented using EchoPlaque 4.0 (INDEC Medical Systems, Los Altos, CA, USA) (Fig. 2). Finally, after injecting an iodinated contrast media (37%), the bifurcation models were imaged with µCT (Skyscan 1172 version 1.5, Antwerp, Belgium) with the following parameters: image pixel size 26.94 µm, voltage 100 kV, current 100 µA, and slice thickness 27 µm. The bifurcations were 3D reconstructed from µCT images with 3D medical imaging software (Rhino3dmedical, Mirrakoi, Switzerland) and smoothed with modeling software Meshmixer (Autodesk Research, New York, NY, USA).

#### IVUS processing for bifurcation lumen reconstruction

The detailed steps of the bifurcation lumen reconstruction are listed in Fig. 1. We applied Electrocardiogram (ECG)-gating to obtain the IVUS frames at the end-diastolic phase. The frames were uploaded into Grasshopper (GH) 3D, a Rhinoceros plug-in. These frames were then aligned and packaged in a straight line along the central path of the IVUS catheter. The packaged frames were oriented in 3D space by placing them perpendicular to the centerline and registering each frame centroid. The MV and SB frames were then rotated to align with the same bifurcation carina reference to match the relative position. The lofting function of GH created the original surfaces of MV and SB. This process’s detailed steps can be found in our previous work on 3D reconstruction using optical coherence tomography (OCT) images^28^.

**Figure 3:**
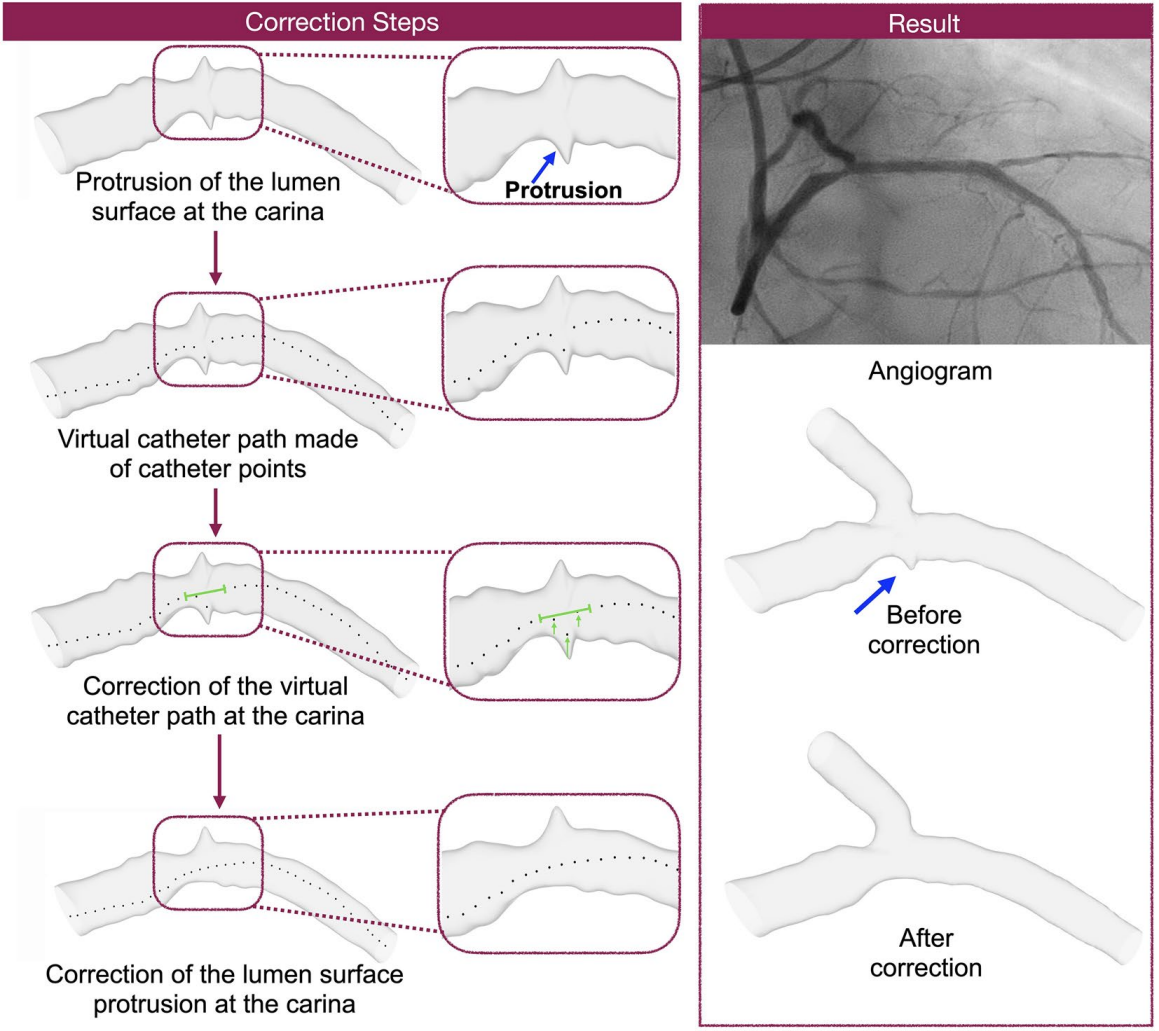
Virtual catheter pathway method to correct surface protrusions near carina during reconstruction.

**Figure 4:**
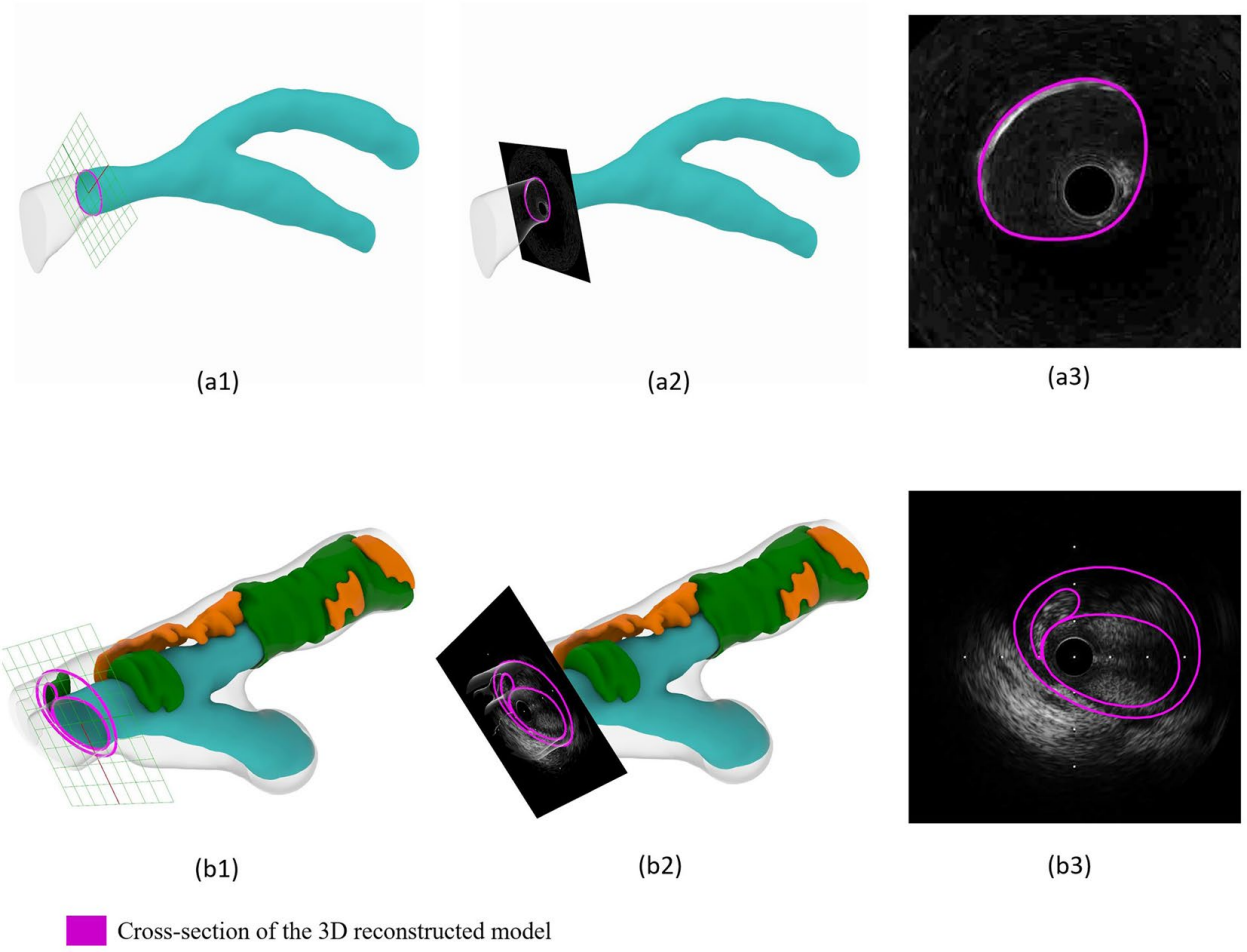
Mapping back technique. (**a**) Silicone model. (**a1**) Cross-section of the 3D reconstructed lumen. (**a2**) Mapping back of the IVUS image to the corresponding 3D reconstructed lumen cross-section. (**a3**) Cross-section view of the mapping-back. (**b**) Clinical case. (**b1**) Cross-section of the 3D reconstructed lumen, calcium, fibrolipid, and fibrosis. (**b2**) Mapping back of the IVUS image to the corresponding 3D reconstructed vessel cross-section. (**b3**) Cross-section view of the mapping-back. IVUS, intravascular ultrasound.

To secure a smooth 3D reconstruction of the MV and SB surfaces, especially at the carina where the MV/SB surfaces exhibited irregular protrusions, in each IVUS frame, we identified the catheter point. All the catheter points of the entire sequence of IVUS frames created a virtual catheter path. We noticed that the catheter points at the carina were deviating. To correct the surface protrusion at the carina, we manually aligned the deviating catheter points (Fig. 3). To further refine the 3D reconstructed lumen surface, we mapped back the segmented IVUS frames to the 3D reconstructed surface (Fig. 4). Using a Rhinoceros tool, named subdivision surfaces (SubD)^29^, we were able to directly adapt the 3D geometry of the lumen to the segmented IVUS frames.

#### Validation of the 3D reconstruction method

The 3D IVUS reconstructed bifurcation models were compared to the corresponding 3D µCT reconstructed ones. The carina point was used to co-register the 3D IVUS and µCT reconstructed models. For comparisons, two metrics were used: lumen diameter and shape. To minimize biases, different operators performed the IVUS-based 3D reconstruction, µCT-based 3D reconstruction, and the comparisons of IVUS- and µCT-based models.

In the IVUS and µCT models, serial cross-sections were identified at every 0.1 mm along the lumen of the MV and SB. We observed a consistent difference in lumen diameter between IVUS and µCT (Supplementary Fig. S1), with IVUS measurements overestimating the actual lumen size^30^. For that reason, the lumen diameters were normalized using the z-score^31^ to account for the systemic discrepancy in lumen size between the IVUS and µCT.

In each cross-section, the ratio of the maximum distance between the two furthest points of the circumference (distance X) to the maximum length perpendicular to distance X (distance Y) was calculated to compare the lumen shape. The lumen was assumed to be oval-shaped, and the ratio of distance Y/distance X was used as a lumen shape indicator.

#### Reproducibility

To calculate the reproducibility of the IVUS-based 3D reconstruction method, two independent operators 3D reconstructed all the silicone models, respectively. The reconstructed models were compared in terms of the lumen diameter and shape.

### Clinical studies

The clinical feasibility and processing times of our bifurcation reconstruction method were evaluated in n = 3 patient coronary artery bifurcations (Supplementary Table S1). The above-mentioned imaging protocols were used to collect IVUS and angiography data. Using a customized platform (based on Rhinoceros and GH), we manually segmented the vessel lumen and EEM, as well as the plaque materials (calcium, fibrosis, and fibrolipid) (Fig. 3). To correct the twisting of the IVUS catheter due to the pulsatile motion of the artery^28^, we rotated the successive IVUS frames (including lumen, EEM, and plaque materials) around the catheter center until the segmented lumens were aligned (Supplementary Fig. S2). Finally, the correctly oriented IVUS frames (lumen, EEM, and plaque materials) were lofted and smoothened to create the 3D bifurcation reconstruction. The mapping back technique was also used to further refine the quality of the 3D reconstructed lumen, EEM, and plaque materials. To evaluate the time efficiency of our 3D reconstruction method, we calculated the processing time for each step in all three clinical cases.

### Statistical analyses

Statistical analyses were performed with the statistical package GraphPad Prism 9.5 (GraphPad Inc., San Diego, CA, USA). Continuous variables were expressed as median (Inter quartile range; IQR). The lumen diameters of IVUS and μCT models were normalized by calculating the Z score as (absolute diameter-µ)/s with µ representing the mean diameter and s the standard deviation of the mean. The comparisons and reproducibility were performed with linear regression and Bland–Altman analysis. p value < 0.01 was considered as the level of significance.

## Results

### Silicone model validation

#### Lumen diameter

**Figure 5:**
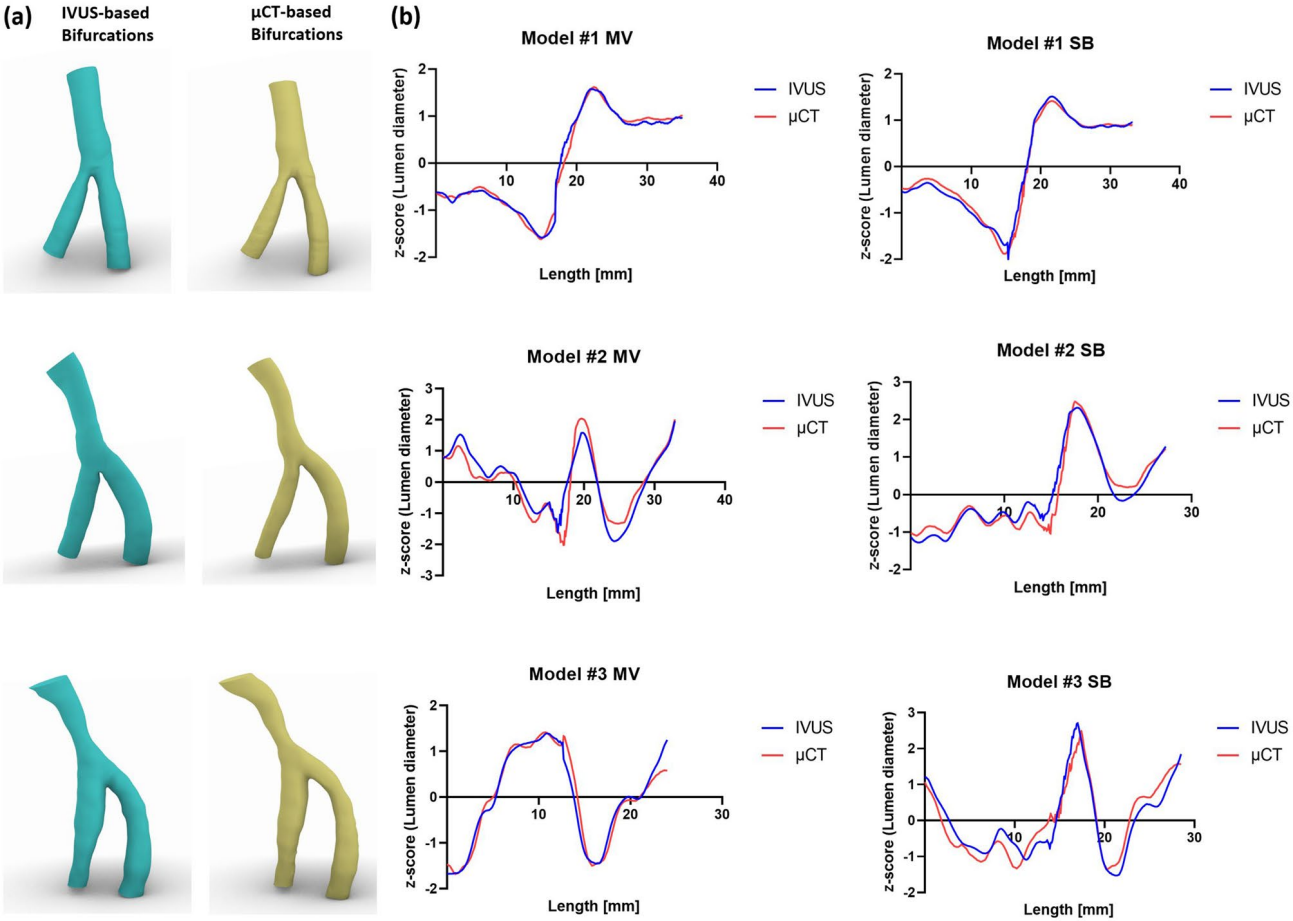
Comparison between IVUS-based and μCT-based 3D reconstruction of silicone bifurcation models. (**a**) IVUS-reconstructed models and μCT-reconstructed models. (**b**) Normalized lumen diameter/length graphs. The lumen length is from distal to proximal. IVUS, intravascular ultrasound; MV, main vessel; SB, side branch.

All n = 3 silicone models were successfully 3D reconstructed (Fig. 5a). In the diameter/length graphs, the normalized lumen diameters (z-score) of the 3D reconstructed bifurcations from 3D IVUS vs. µCT show high agreement (Fig. 5b). Linear regression analysis revealed r^2^ values ranging from 0.88 to 0.99, with slopes close to one and intercepts near zero (Table 1).

**Table 1:** Comparison between IVUS-based and μCT-based 3D reconstructed silicone models.

Bifurcation	Branch	Lumen diameter		Lumen shape			
		r^2^	Linear regression equation	IVUS median	25th, 75th percentile	µCT median	25th, 75th percentile
#1	MV	0.99	y = 1.00x + 0.00	0.92	0.89, 0.93	0.94	0.91, 0.96
	SB	0.99	y = 0.99x + 0.00	0.92	0.90, 0.94	0.95	0.92, 0.96
#2	MV	0.88	y = 0.90x−0.03	0.90	0.85, 0.93	0.91	0.86, 0.95
	SB	0.94	y = 0.97x + 0.00	0.87	0.72, 0.93	0.84	0.72, 0.91
#3	MV	0.96	y = 0.98x + 0.00	0.84	0.80, 0.92	0.83	0.74, 0.92
	SB	0.88	y = 0.94x + 0.00	0.90	0.85, 0.95	0.90	0.78, 0.94

Linear regression analysis of the normalized lumen diameters (z-score) and median with interquartile range for lumen shape.

IVUS, intravascular ultrasound; µCT, micro-computed tomography; MV, main vessel; SB, side branch.

#### Lumen shape

Table 1 shows the median, 25th, and 75th percentiles of the maximum distances perpendicular to each other (distance Y/distance X) of IVUS- and µCT-reconstructed bifurcation models. The median, 25th, and 75th percentile ratios of reconstructed IVUS and µCT models revealed mean differences of − 0.003 (0.046 to 0.039), 0.013 (− 0.067 to 0.093), and − 0.007 (− 0.045 to 0.032), indicating a high level of agreement (Supplementary Fig. S3).

#### Reproducibility

Table 2 displays the reproducibility test results for our method. The lumen diameters of the 3D IVUS reconstructed bifurcation models from two independent operators showed high agreement, with r^2^ ranging from 0.96 to 1.00 (p < 0.0001), indicating that our method was quite reproducible.

**Table 2:** Inter-observer reproducibility of the proposed algorithm.

Bifurcation	Branch		Lumen diameter	
		r^2^	Linear regression equation	p value
#1	MV	1.00	y = 1.01x−0.05	< 0.0001
	SB	0.96	y = 0.99x+0.10	< 0.0001
#2	MV	0.98	y = 1.00x+0.00	< 0.0001
	SB	1.00	y = 1.01x−0.02	< 0.0001
#3	MV	0.99	y = 1.02x−0.03	< 0.0001
	SB	0.99	y = 1.04x−0.12	< 0.0001

MV, main vessel; SB,  side branch.

### Clinical feasibility

**Figure 6:**
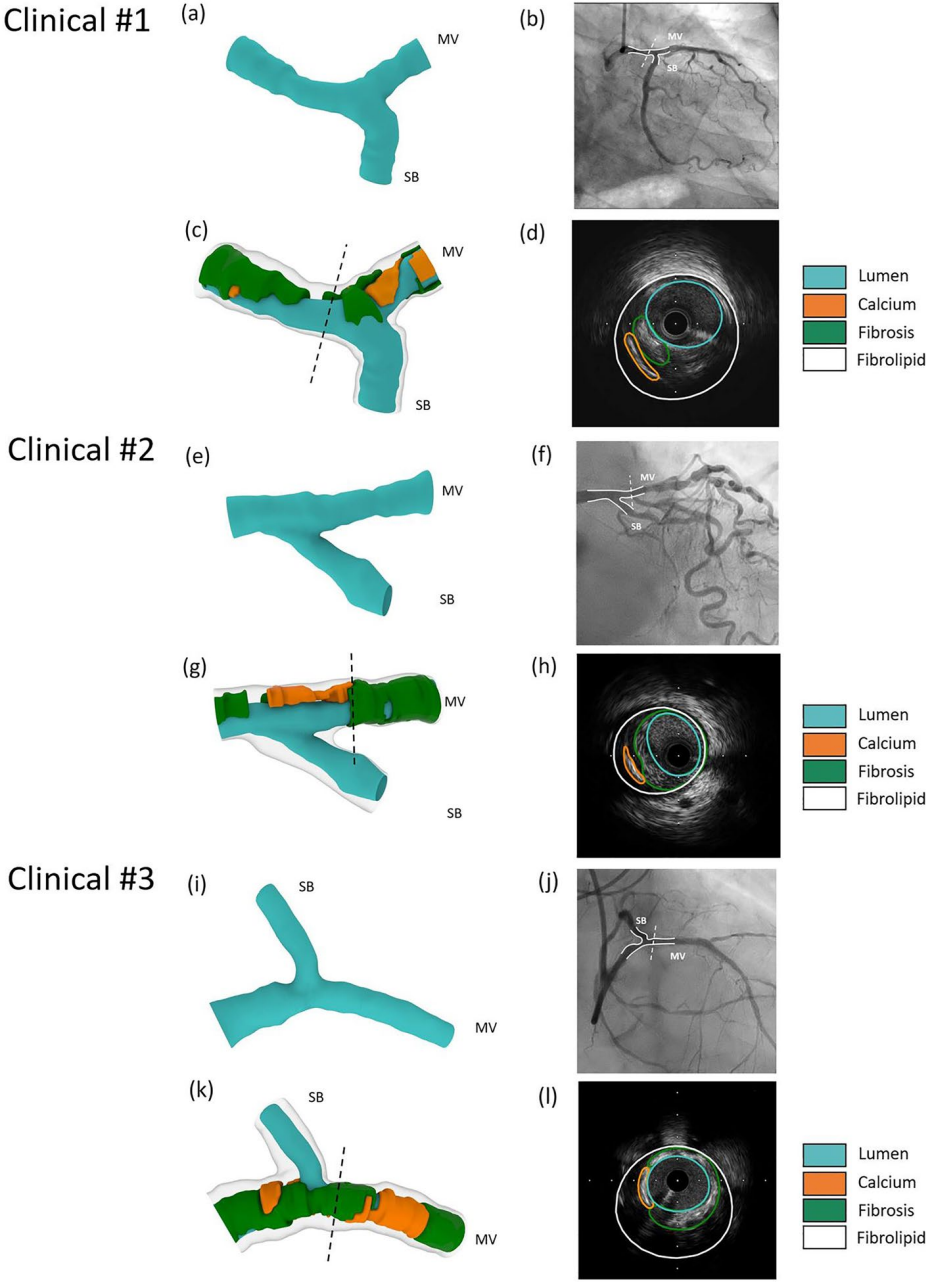
3D reconstruction of coronary artery bifurcation geometries. (**a**, **e**, **i**) Reconstructed lumen of n = 3 patient-specific geometries using angiography and IVUS imaging. (**b**, **f**, **j**) Angiography of the reconstructed coronary bifurcation lumen showing the main vessel (MV) and side branch (SB). (**c**, **g**, **k**) Reconstructed lumen, calcium, fibrosis, and fibrolipid of n = 3 patient-specific geometries using angiography and IVUS imaging. (**d**, **h**, **l**) Mapping back of a cross-section of the reconstructed lumen, calcium, fibrosis, and fibrolipid to its respective IVUS image, showing the high accuracy of the reconstruction. IVUS, intravascular ultrasound; MV, main vessel; SB, side branch.

The validated 3D reconstruction algorithm successfully reconstructed all n = 3 patient bifurcations. The reconstructed bifurcation models, including lumen, EEM, and plaque materials, are shown in Fig. 6. The lumen shape was in good agreement with the corresponding angiograms (Fig. 6b, f, j). The mapping back technique was used in all models to test the accuracy of the plaque materials reconstruction, showing that the reconstructed lumen and plaque materials matched their respective IVUS images in location and size (Fig. 6d, h, l).

Table 3 summarizes the processing time for each step, from image processing to final 3D lumen and plaque reconstruction. The average time for image pre-processing was about 100 min, and for 3D reconstruction of a bifurcation about 60 min.

**Table 3:** Processing times for the IVUS-based 3D reconstruction of patient-specific coronary artery bifurcations (n = 3).

Steps	Minutes
Step 1. Image pre-processing	
1. Angiography processing	15 ± 10
2. IVUS segmentation	85 ± 15
Total time for image pre-processing	100
Step 2. 3D reconstruction of bifurcation lumen	
1. Data importing and parameter setting	20 ± 5
2. IVUS frame error correction	2 ± 1
3. Localization and rotation of IVUS frames on the centerline	2 ± 1
4. 3D reconstruction of MV/SB models	2 ± 1
5. 3D reconstruction of plaques and final model	30 ± 5
Total time for 3D reconstruction of bifurcation lumen	56
Total time for the whole process	156

IVUS, intravascular ultrasound; MV, main vessel; SB, side branch.

## Discussion

In this study, we proposed a new method for 3D reconstruction of coronary bifurcations, including plaque materials, by combining IVUS imaging and coronary angiography. We performed in-vitro validation using silicone models and clinical feasibility tests, demonstrating that our reconstruction technique has high accuracy, reproducibility, and clinical feasibility. Our validated method has the potential to be used in the clinical setting to provide reliable information about the anatomy and plaque burden of coronary bifurcations, thereby facilitating clinical planning and decision-making during cardiac catheterization procedures.

The first studies on IVUS-based coronary vessel 3D reconstruction date back more than 20 years^5–7^. Thereafter, several studies have performed 3D reconstruction of non-bifurcated coronary arteries by combining IVUS with angiography^5–23^. To date, two studies have performed 3D reconstruction of coronary bifurcation arteries by combining IVUS and angiography, but both studies used CT to correct the IVUS frames’ orientation^24,25^. Our study is the first to perform 3D reconstruction of coronary artery bifurcation by combining IVUS and angiography only without the need for CT imaging. While some previous studies have performed 3D reconstruction of coronary plaque materials based on IVUS, they have certain limitations. Balocco et al.^21^ performed segmentation and reconstruction of fibrotic, calcified, and fibro-lipidic plaques considering thickness, but simplified the models by assigning plaques’ morphological values to vessel surface mesh. By collecting the IVUS-VH (Virtual Histology) cross-section image, Liang et al.^26^ reconstructed fibrous tissue, necrotic core, and calcification for single vessels. Following the 3D reconstruction methods proposed by Tang et al.^32^, a series of works applying IVUS or IVUS-VH were performed by Tang’s research group^22,23,27^. They reconstructed 3D models of fibrous tissue, lipid core, and calcification but the reconstructions were limited to non-bifurcated vessels. Compared to these prior works, our study provides a more comprehensive 3D bifurcation reconstruction incorporating all major coronary plaque materials: calcium, fibrosis, and fibro-lipid. Table 4 provides a comprehensive comparison of our methodology to that of other published studies.

**Table 4:** Literature summary of the IVUS-based methods of 3D reconstruction of coronary arteries.

Study	Study data	No. of cases	Imaging modality	Segmentation technique	3D reconstruction technique	Processing time	Bifurcation included	Plaque materials reconstruction	Validation method	Clinical feasibility	Limitations
Wu et al. (current study)	Bench Clinical	6 arteries (3 bench and 3 clinical)	IVUS + Angiography	Manual segmentation (lumen, EEM, plaque materials)	3D centerlines of MS and SB; virtual catheter pathway; Reconstruction of plaque materials; Mapping back of reconstruction	< 1 h for reconstruction	Yes	Yes	In-vitro—compared to the μCT-based model Clinical—compared to angiography and mapping back to IVUS	Yes	1. The study had a small sample size 2. IVUS and µCT 3D reconstructed model’s lumen diameter comparison using z-score normalization 3. Swinging effect was not taken into consideration 4. Long manual IVUS image segmentation time
Wahle et al. (1999)^5^	Bench Animal	3 arteries (1 silicone model and 2 cadaveric pig RCA)	IVUS + Angiography	Automatic segmentation (lumen and EEM)—Automatic segmentation + Manual correction	3D trajectory reconstruction using Angiogram, followed by 3D matching of IVUS frames, estimation of relative twist, followed by 3D mapping of contours and estimation of absolute orientation	N/A	No	No	In-vitro (silicone models)—computational simulation	No	1. High variance in the pullback speed during manual pullback, leading to significant errors in the matching of estimated frame locations, and calculations of the local axial twist 2. The approach was not applicable to clinical cases as they did not consider the problems with ECG and respiratory gating
Cothren et al. (2000)^6^	Clinical Animal	11 arteries (5 human and 6 pig) 11 LAD	IVUS + Angiography	Automatic segmentation (lumen and EEM)	Biplane angiography—3D IVUS catheter path determined; IVUS—lumen and EEM contour determined; Fusion of IVUS and angiographic image to get IVUS image on 3D catheter path. Correction of rotational orientation	N/A	No	No	Ex-vivo—Self-validation, 5 LAD coronary arteries were reconstructed twice in the same co-ordinate system from 2 independent and different pairs of angiographic planes	No	1. Manual gating of all IVUS images using ECG signals increased the time required to perform the reconstruction 2. Manual pullback was performed, which resulted in irregular pullback speed, unstable catheter path and several distortions 3. The study assumed that the artery does not move between its two reconstructions. Despite care, some tissue movement always occurred while inserting the catheter in the artery after the first IVUS pullback, introducing error
Slager et al. (2000)^7^	Bench Clinical	14 arteries (2 bench and 12 clinical) 7 RCA 4 LAD 1 LCX	IVUS + Angiography	Semi-automatic segmentation	Biplane angiography—3D IVUS catheter path determined; IVUS—lumen and EEM contour determined; Fusion of IVUS and angiographic image to get IVUS image on 3D catheter path. Correction of rotational orientation	N/A	No	No	In-vivo + ex-vivo—validated against in-vitro method, both reconstructed using IVUS + Angiography	Yes	1. The validation method was restricted in rather short arterial segments including stents
Bourantas et al. (2003)^8^	Bench Clinical Animal	10 cases for experiment 27 cases for validation (21 human and 6 sheep)	IVUS + Angiography	Semi-automatic segmentation (lumen and EEM)	Biplane angiography—Catheter path extraction; IVUS—detection of lumen and EEM border; placement of IVUS frame on catheter path and estimation of relative axial twist; Orientation of reconstructed vessel based on absolute orientation of the first IVUS frames	N/A	No	No	1. Separate validation of each step 2. Catheter path extraction method—11 patient data set (in-vivo) + phan-tom catheter/wire model (in-vitro) 3. Semiautomated border detection algorithm—10 patient data set (in-vivo) 4. Algorithms used to orient IVUS borders onto the 3D path—6 cadaveric sheep hearts (ex-vivo)	No	1. 1^st^ frame borders were segmented manually. Successive frames were segmented automatically using the borders obtained from the previous processed frames as an initial guess 2. The validation methodology was only able to estimate the accuracy of each individual stage, but not the overall method performance 3. No evaluation of in-vivo factors affecting the triangulation algorithm: patient movement, blood pressure changes, and breathing, which can affect IVUS catheter twist behavior
Bourantas et al. (2005)^9^	Bench Clinical Animal	10 cases for experiment 27 cases for validation (21 human and 6 sheep)	IVUS + Angiography	Semi-automatic segmentation (lumen and EEM)	Biplane angiography—Catheter path extraction; IVUS—detection of lumen and EEM border; placement of IVUS frame on catheter path and estimation of relative axial twist; Orientation of reconstructed vessel based on absolute orientation of the first IVUS frames	N/A	No	No	1. Separate validation of each step 2. Catheter path extraction method—11 patient data set (in-vivo) + phan-tom catheter/wire model (in-vitro) 3. Semiautomated border detection algorithm—10 patient data set (in-vivo) 4. Algorithms used to orient IVUS borders onto the 3D path—6 cadaveric sheep hearts (ex-vivo).	No	1. Non-sheathed IVUS catheter were used, and it was presumed that the catheter tip followed the direction of the catheter during the pull-back, ignoring the lateral movements of the catheter 2. The triangulation algorithm's effectiveness was uncertain due to potential errors in catheter path reconstruction and metal clip placement during experiments on cadaveric sheep hearts 3. No evaluation of in-vivo factors affecting the triangulation algorithm: patient movement, blood pressure changes, and breathing, which can affect IVUS catheter twist behavior
Giannoglou et al. (2006)^10^	Clinical	17 arterial segments RCA = 7 LAD = 3 LCX = 6 Diagonal = 1	ECG gated IVUS image + Angiography	Semi-automatic segmentation (lumen and EEM)	Angiography—Catheter path extraction; IVUS—semi-automated segmentation of lumen and EEM border; Localization of IVUS frame on catheter path; Estimation of relative axial twist; Back projection on angiographic planes; Absolute Orientation of reconstructed vessel	180 min	No	No	In-vivo—qualitative evaluation—Each reconstructed lumen was back-projected onto both angiographic planes and compared quantitatively with the angiographic luminal outlines Quantitative evaluation—all the distances and diameters of the 17 reconstructed lumens were concentrated in a data pool and compared with the corresponding values of the angiographic lumens	Yes	1. The study assigned the lumen and media-adventitia contours equidistantly on the catheter path instead of placing each one at a specific location 2. The reconstruction process of a single vessel limited to EEM and lumen took 3 h 3. Validation method was limited by the twisting motion of the IVUS catheter during the pullback and the geometrical distortions due to cardiac and respiratory movements. Manual gating of IVUS images on ECG was performed to eliminate these distortions
Bourantas et al. (2008)^11^	Bench Clinical Animal	27 arteries	IVUS + Angiography	Semi-automatic segmentation (lumen and EEM)	Biplane angiography—Catheter path extraction; IVUS—detection of the lumen and EEM border; placement of IVUS frame on catheter path and estimation of relative axial twist; Orientation of reconstructed vessel based on the absolute orientation of the first IVUS frames	For a typical luminal narrowing of 25 mm, the total time for reconstruction was approximately 10 min	No	EEM and simple plaque	1. Separate validation of each step 2. Catheter path extraction method—11 patient data set (in-vivo) + phan-tom catheter/wire model (in-vitro) 3. Semiautomated border detection algorithm—10 patient data set (in-vivo) 4. Algorithms used to orient IVUS borders onto the 3D path—6 cadaveric sheep hearts (ex-vivo)	Yes	1. ANGIOCARE failed to reconstruct segments with very narrow lumen or severe stenosis 2. The validation methodology was only able to estimate the accuracy of each individual stage, but not the overall method performance 3. The triangulation algorithm's effectiveness was uncertain due to potential errors in catheter path reconstruction and metal clip placement during experiments on cadaveric sheep hearts
Chatzizisis et al. (2008)^12^	Clinical	16 arterial segments RCA = 6 LAD = 4 LCX = 6	IVUS + Angiography	Semi-automatic segmentation (lumen and EEM)	Angiography—Catheter path extraction; IVUS- semi-automated segmentation of lumen and EEM border; Placement of IVUS frame on catheter path; Estimation of relative axial twist; Connecting corresponding borders of adjacent IVUS images using b-spline curves to build real artery geometry; Absolute Orientation of reconstructed vessel	N/A	No	No	No	Yes	1. The applicability of the method was limited to IVUS imaging systems utilizing sheath-based catheters, which secure a steady pullback trajectory 2. Inability to incorporate side branches in the reconstruction does not allow complete vessel reconstruction
Schuurbiers et al. (2009)^13^	Clinical	10 arteries RCA = 5 LAD = 2 LCX = 3	ECG gated IVUS image + Angiography	Semi-automatic segmentation (lumen and EEM)	Biplane angiography using dilute contrast—3D catheter path determined; IVUS—lumen and stent contour determined, fused with 3D catheter path; Correction of angular rotation	N/A	No	No	In-vivo—Comparison by reconstruction of same vessels using CAAS QCA-3D	Yes	1. The study had a small sample size 2. Diluted contrast agent was used for the biplane angiograms, reducing the image quality of the angiograms. This led to a greater intra-and inter-observer variability 3. IVUS technique used cross-sections perpendicular to the IVUS catheter to determine the lumen area, while CAAS QCA-3D took cross-sections perpendicular to the lumen centerline introducing potential error
Bourantas et al. (2013)^14^	Clinical	22 arteries 7 LAD 7 LCX 8 RCA	IVUS + Angiography	Semi-automatic segmentation (lumen and EEM)	Biplane angiography—3D Lumen centerline estimation; IVUS—detection of lumen and EEM border; placement of IVUS frame on 3D centerline and estimation of relative axial twist using sequential triangulation algorithm; Projection of reconstructed vessel onto angiographic images; Orientation of reconstructed vessel based on absolute orientation of the first IVUS frames; Construction of two NURBS and rebuilding of lumen and EEM 3D geometry	N/A	No	No	Self-Validation by comparing with their conventional reconstruction method explained	Yes	1. Time consuming reconstruction process 2. Axial twist of the IVUS catheter occurred during its pullback leading to relative twist of the IVUS frames. This was estimated using of the sequential triangulation algorithm
Doulaverakis et al. (2013)^15^	Clinical	31 human coronary arteries RCA = 11 LAD = 8 LCX = 12	ECG gated IVUS + Angiography	Semi-automatic or fully automatic segmentation (lumen and EEM)	Biplane angiography—Catheter path extraction; IVUS—automatic/semi-automatic detection of lumen and EEM border; placement of IVUS contour on 3D catheter path and estimation of relative orientation of IVUS contours; Absolute Orientation of reconstruction by back-projections of the reconstructed lumen to the angiography planes; Generation of lumen and EEM geometries using NURBS surfaces	Automatic IVUS processing = 52.4 ± 14.6 min Automatic IVUS processing with manual correction = 115.5 ± 33.6 min Semi-automatic IVUS processing = 134.9 ± 39.5 min	No	No	Separate validation of each step 1. Automated process of ECG gating was validated against an operator dependent manual ECG gating 2. Automatic /semi-automatic segmentation was validated against manual segmentation 3. Time taken for automatic and semi-automatic reconstruction against manual processing	Yes	1. The 3D reconstruction results were based on a single-user experience of the software; no inter-observer variability comparison performed 2. The automatic segmentation algorithms are amenable to further improvement 3. The validation methodology was applied to estimate the accuracy of each individual stage, but not the overall method performance
Ma et al. (2013)^16^	Clinical	N/A	IVUS + Angio	Semi-automatic segmentation (lumen and EEM)	Single plane angiography—3D vessel centerline reconstruction; Preprocessing of IVUS images; IVUS—detection of lumen and EEM border; IVUS frame placed on 3D centerline and relative axial twist calculated; Vessel EEM and lumen surface reconstruction based on NURBS surfaces. Projection of reconstructed vessel onto angiographic plane	N/A	No	No	N/A	No	1. The method was not validated by comparing the reconstruction with another methodology or imaging technique 2. No inter-observer variability comparison performed 3. The automatic segmentation algorithms are amenable to further improvement
Zheng et al. (2013)^17^	Clinical	5 coronary arteries	ECG gated IVUS image + Angiography	Semi-automatic segmentation (lumen and EEM)	Biplane angiography—Catheter path extraction; IVUS—detection of lumen and EEM border; Placement of IVUS contour on 3D catheter path and estimation of relative orientation of IVUS contours; Generation of lumen and EEM geometries using NURBS surfaces; Absolute Orientation of reconstruction by back-projections of the reconstructed lumen along the 3-D catheter pullback path	N/A	No	No	In-vitro and in-vivo data validation— 1. Computer simulations—determination of relative catheter twist or spatial orientation of IVUS frames 2. back-projection validation—reprojecting the 3D vessel model onto angiographic image planes 3. Quantitative measurement of morphological parameters like vessel segment length, volume, and the plaque volume	Yes	1. In-vivo validation was performed in a small series of data (Five pullback data sets) 2. Motion artifact, catheter-related artifact, and rotation angle artifact, present in intracoronary ultrasound images, might have resulted in vessel deformation in acquired cross-sectional images and longitudinal cuts 3. The research mainly focused on the reconstruction at end-diastolic phase. This limitation reduced the potential of IVUS in coronary arteries for studying bio-mechanical properties and evaluating the vessel EEM shear stress
Gijsen et al. (2014)^24^	Clinical	10 coronary arteries RCA = 1 LAD = 8 LCX = 1	IVUS + Multislice CT (MSCT)	Semi-automatic segmentation (lumen and EEM)	3D reconstruction of the main branch—3D centerlines obtained from MSCT data set. Curved multi planar reconstructions (MPR) generated perpendicular to the centerline. IVUS contour stacks placed perpendicular to centerlines Proximal part of the main branch and the side branch—MSCT based centerlines extraction. MPR generated. Lumen boundary of prox. MV and SB manually delineated in longitudinal plane. Cross-sectional contours extracted Merging of 3D data sets from step 1 and 2 using in-plane scaling of MSCT contours to IVUS segmentation Generation of bifurcation geometry—Proximal SB reconstructed either by combining MSCT and IVUS or MSCT only. Distal SB constructed using MSCT	N/A	Yes	No	Self-validation by comparing reconstruction using MSCT only and MSCT + IVUS	Yes	1. SB with a diameter < 0.5 mm was not reconstructed. Reconstruction procedures depended on MSCT data for SB reconstruction. MSCT has limited and inferior spatial and temporal resolution. MSCT may not accurately identify small SBs, hampering the reconstruction process 2. Calcifications induce blooming artifacts in MSCT which can reduce the accuracy of the lumen segmentation 3. The shear stress distribution in the bifurcation region is influenced by the outflow boundary conditions, especially the flow distribution between the MV and the SB important. This study applied the same outflow conditions for the two reconstruction procedures, thus the choice for the flow distribution did not influence the shear stress maps comparison in the bifurcation regions
Bezerra et al. (2015)^18^	Clinical	N/A	ECG gated IVUS + Angiography	Semi-automatic segmentation (lumen and EEM)	Angiography—3D vessel centerline reconstruction; Preprocessing of IVUS images; IVUS—detection of lumen and EEM border; defining position of each frame on catheter trajectory; Placement of IVUS frame perpendicular to catheter trajectory and estimation of relative axial twist	N/A	No	No	No	No	1. The movement of the IVUS catheter and vessel curvature hampered the volumetric estimation of plaque burden 2. The presence of stenosis resulted in disagreement between center lines of the vessel lumen and EEM (asymmetry) 3. The method was not validated by comparing the reconstruction with another methodology or imaging technique
Son et al. (2017)^25^	Bench	N/A	IVUS + Angiography + CT scan	Manual segmentation (lumen and EEM)	CT Scan—Undeformed intima, centerline, intimal cross-section; Angiography—3D Catheter path; Angiographic 3D catheter path + IVUS—deformed intimal/adventitial model, 3D centerline from IVUS + angiography, intima/adventitia cross-section; 3D reconstruction by cross-registration of IVUS + Angiographic deformed intima/adventitia and CT undeformed intima	N/A	Yes	No	Cross registration between reconstructed deformed vessel intima/adventitia obtained from IVUS + Angiography and undeformed vessel intima reconstructed using CT	No	1. IVUS cross sections were registered with CT intima cross sections. The movement, rotation, and scale values calculated through registration between the two intima cross sections were equally applied to the adventitia extracted from IVUS images, which produced errors 2. The method calculated an ideal result without considering the material properties of the blood vessel which can deform the intima-adventitia calculation 3. It was difficult to accurately evaluate the accuracy of the blood vessel model as adventitia information was only based on IVUS images
Wang et al. (2018)^19^	Clinical	22 stenotic arteries 17 LAD 1 LCX 3 RCA 1 LM	IVUS + Angiography	Semi-automatic segmentation (lumen and EEM)	Angiography—Edge detection, 3D catheter path reconstruction; Localization and Orientations of IVUS Frames—Relative orientation between consecutive IVUS frames, followed by absolute orientation of the whole IVUS image sequence	N/A	No	No	Comparison of the results of correlation of the reconstructed 3-D catheter trajectory back-projected to the XOZ (RAO), YOZ (LAO) plane with the angiographic planes of the 2-D catheter guide wire	Yes	1. Single stenotic branches were reconstructed for hemodynamics analysis. SBs were not included 2. Small Sample size 3. Findings required further analysis to validate their coronary models for hemodynamic analysis of stenotic artery
Jiang et al. (2021)^20^	Clinical	32 arteries RCA = 6 LAD = 25 LCX = 1	IVUS + Angiography	Automatic IVUS segmentation	3D catheter path reconstruction; IVUS—diastolic phase image selection, automatic segmentation of lumen and EEM contour; Stacking IVUS segmented contours perpendicular to the catheter path/trajectory. Correction of relative twist followed by definite/absolute orientational correction	N/A	No	No	N/A	Yes	1. The research method was validated using only (29) vessels, and no left main arterial lesions were included in the analysis 2. Automatic IVUS segmentation was based on deep machine learning which has unproven accuracy for IVUS segmentation
Yang et al. (2009)^23^	Clinical	1	IVUS-VH + Angiography	Automatic IVUS segmentation	Automated contour generation using in-house software (APIA). Smoothing and preparation of contour plots for model construction. Removal of isolated small components and combining close components. Integration of X-ray angiographic data for determination of stenosis segment movement and curvature. Preprocessing of 3D data using VTK. Identification and inclusion of plaque components. Construction of fitting areas and volume stacking. Generation of computational mesh using ADINA	N/A	No	Yes	N/A	Yes	1. Lack of biplane angiography that could be used to reconstruct 3D vessel curvature 2. Patient-specific and tissue-specific material properties were not available for the study 3. The in-vivo study was unable to measure multi-layer vessel morphology and material non-invasively
Wang et al. (2015)^22^	Clinical	1	IVUS-VH + Angiography	Automatic IVUS segmentation	Automated contour generation using in-house software (APIA). Smoothing and preparation of contour plots for model construction. Removal of isolated small components and combining close components. Integration of X-ray angiographic data for determination of stenosis segment movement and curvature. Preprocessing of 3D data using VTK. Identification and inclusion of plaque components. Construction of fitting areas and volume stacking. Generation of computational mesh using ADINA	N/A	No	Yes	N/A	Yes	1. Lack of biplane angiography that could be used to reconstruct 3D vessel curvature 2. Patient-specific and tissue-specific material properties were not available for the study 3. The in-vivo study was unable to measure multi-layer vessel morphology and material non-invasively
Balocco et al. (2012)^21^	Clinical	10	IVUS + Angiography	Automatic IVUS segmentation	Assessment of vessel morphology by coupling IVUS with angiographic projections. Establishing a global reference system using Angio-1 and Angio-2. Reconstruction of the catheter path and fitting a spline to the data. Estimating the angular rotation of the local reference system using a bifurcation path and manual identification of torsion. Compensation for catheter rotations and alignment of successive IVUS frames. Generation of triangular mesh for vessel surface. Sampling and connecting contour points. Assignment of morphological values to vessel surface and volumetric mesh	N/A	No	Yes	N/A	Yes	1. Single vessel reconstruction without validation 2. The plaque material properties are “mapped” to surface, lacking real 3D configurations
Liang et al. (2014)^26^	Clinical	4	IVUS-VH	Automatic IVUS segmentation	IVUS-VH cross-sections across catheter center path. Each tissue surface generated. Automated plaque material boundaries generated. The 3D geometry converted to triangular finite elements mesh	N/A	No	Yes	N/A	Yes	1. Single vessel reconstruction 2. The arterial wall was modeled as a uniform entity. Thus, layer-specific modeling of the vessel components was absent 3. Method did not use angiography to get the correct catheter path
Fan et al (2014)^27^	Clinical	10	IVUS + Angiography	Automatic IVUS segmentation	Automated contour generation using in-house software (APIA). Smoothing and preparation of contour plots for model construction. Removal of isolated small components and combining close components. Integration of X-ray angiographic data for determination of stenosis segment movement and curvature. Preprocessing of 3D data using VTK. Identification and inclusion of plaque components. Construction of fitting areas and volume stacking. Generation of computational mesh using ADINA	N/A	No	Yes	N/A	Yes	1. Lack of biplane angiography that could be used to reconstruct 3D vessel curvature 2. Patient-specific and tissue-specific material properties were not available for the study 3. The in-vivo study was unable to measure multi-layer vessel morphology and material non-invasively

Angio, angiography; ECG, electrocardiography; CT, computed tomography; μCT, micro-computed tomography; MSCT, multislice computed tomography; IVUS, intravascular ultrasound; IVUS-VH, intravascular ultrasound Virtual Histology; QCA, quantitative coronary angiography; MV, main vessel; SB, side branch; RCA, right coronary artery; LM, left main coronary artery; LAD, left anterior descending; LCX, left circumflex artery; NURBS, non-uniform rational B-spline surfaces; EEM, external elastic membrane.

We validated our work using 3D models reconstructed from µCT imaging, widely accepted as a gold standard^33,34^, after successfully reconstructing patient-specific silicone bifurcation models based on IVUS and angiography. The linear regression analysis on lumen diameter revealed that the r^2^ values ranged from 0.88 to 0.99, with a mean difference of -0.003 in the median lumen shape ratio. The linear regression analysis of the reproducibility test, performed by two independent operators, revealed high r^2^ values ranging from 0.96 to 1.00, suggestive of the high accuracy and reproducibility of the proposed method. During the clinical feasibility tests, the mapping back of the IVUS images onto the corresponding 3D reconstructed model demonstrated that our 3D reconstruction method could accurately locate and size the plaque materials.

Notably, in our work, we used two innovative techniques: (1) creation of the virtual catheter path to correct the irregular surface protrusion at the carina, and (2) mapping back technique combined with the SubD surfaces, which allowed us to directly modify and adapt the 3D reconstructed bifurcation model to the segmented IVUS frames (Supplementary Fig. S4).

This study’s findings have important clinical implications and future applications. Our 3D reconstruction method has the potential to improve procedural planning and clinical outcomes by providing interventional cardiologists with precise information about the anatomy and severity of coronary bifurcation disease. Our technique can also perform patient-specific computational simulations for bifurcation stenting. This can advance knowledge about bifurcation stenting mechanics and potentially lead to more personalized stenting techniques. This can be especially beneficial to the pharmaceutical industry when developing next-generation stents. Furthermore, if the imaging data is available to extract the required information, the innovative methodology of our technique has the potential to improve other invasive imaging modalities, such as OCT, or even non-invasive imaging, such as coronary computed tomography angiography. Finally, when combined with improved visualization techniques, the accurate 3D reconstructed bifurcation models can provide medical students with novel education, such as flying through the vessel using virtual reality.

## Limitations

There were several limitations in our study. First, our study was performed on a small dataset (three silicone models and three clinical cases). However, the primary goal of our proof-of-concept study was to introduce a novel 3D reconstruction method by IVUS and angiography, validate this with silicone models, and show its clinical applicability. Second, the back-and-forth movement of the IVUS catheter (swinging effect) during pulsation could affect the accuracy of the 3D reconstruction^35^. However, the application of ECG-gating could minimize the swinging effect, as suggested by the previous works. Finally, manual segmentation of IVUS images and plaque material is a time-consuming process. To address this issue, we are currently working on machine learning-based codes that can be integrated into GH to fully automate the IVUS segmentation process, reducing processing time and allowing our algorithm to be applied in near real-time.

## Conclusion

This study presents a new methodology for 3D reconstruction of coronary artery bifurcations using IVUS and angiography. The agreement between the IVUS-based 3D reconstructed bifurcation models and the µCT reference models was remarkably high, indicating the accuracy of our approach. Furthermore, our methodology was found to be clinically feasible and time-efficient for three patient-specific bifurcations, with a reconstruction time of about 60 min. Our technique provides a simple, easy-to-use, and accurate 3D reconstruction of coronary bifurcations, which can help with pre-procedural planning and clinical decision-making for bifurcation stenting procedures.

### Supplementary Information

Supplementary Information.

## Data Availability

Correspondence and requests for materials should be addressed to Y.S.C.
