# Author Correction: 3D reconstruction of coronary artery bifurcations from intravascular ultrasound and angiography

**DOI:** 10.1038/s41598-024-57034-w

**Published:** 2024-03-19

**Authors:** Wei Wu, Usama M. Oguz, Akshat Banga, Shijia Zhao, Anjani Kumar Thota, Vinay Kumar Gadamidi, Charu Hasini Vasa, Khaled M. Harmouch, Abdallah Naser, Xiarepati Tieliwaerdi, Yiannis S. Chatzizisis

**Affiliations:** 1 Center for Digital Cardiovascular Innovations, Division of Cardiovascular Medicine, Miller School of Medicine, University of Miami, Miami, FL USA; 2 Division of Cardiovascular Medicine, Leonard M. Miller School of Medicine, University of Miami Health System, University of Miami, 1120 NW 14Th Street, Suite 1124, Miami, FL 33136 USA

Correction to: *Scientific Reports* 10.1038/s41598-023-40257-8, published online 10 August 2023

The original version of this Article contained errors.

In the Methodology section, incorrect information was provided regarding the source of patient data and ethical approval of the study protocol.

“All methods were carried out in accordance with the relevant guidelines and regulations. The angiograms and IVUS data used in the study were obtained from a clinical trial named PROPOT (Randomized Trial of the Proximal Optimization Technique in Coronary Bifurcation Lesions). The study was reviewed and approved by the ethics committee of Teikyo University (IRB approval number 15-159-2), and informed consent was obtained from all participants.”

now reads:

“All methods were carried out in accordance with the relevant guidelines and regulations. The angiograms and IVUS data used in the study were obtained from the Kyushu Medical Center, Fukuoka, Japan. The study was reviewed and approved by ethics committee of the National Hospital Organization Kyushu Medical Center (protocol number 20C035). Informed consent was obtained from all participants.”

The original Article has been corrected.

