# Supplementary material for: 3D reconstruction of coronary artery bifurcations from intravascular ultrasound and angiography
Source: Sci Rep. 2023 Aug 10;13:13031. doi: 10.1038/s41598-023-40257-8 (PMC10415353; doi:10.1038/s41598-023-40257-8)
Supplement: Supplementary file 1 — Supplementary Information. [file 41598_2023_40257_MOESM1_ESM.docx]

**3D Reconstruction of Coronary Artery Bifurcations from** **Intravascular Ultrasound and Angiography**

Wei Wu, PhD, Usama M. Oguz, MD, Akshat Banga, MBBS, Shijia Zhao, PhD, Anjani Kumar Thota, MBBS, Vinay Kumar Gadamidi, MD, Charu Hasini Vasa, MBBS, Khaled M. Harmouch, MD; Abdallah Naser, MD; Xiarepati Tieliwaerdi, MD; Yiannis S. Chatzizisis, MD, PhD

Center for Digital Cardiovascular Innovations, Division of Cardiovascular Medicine, Miller School of Medicine, University of Miami, Miami, Florida, United States of America

**Funding:** Supported by the National Institute of Health (R01 HL144690) and Dr. Vincent Miscia Cardiovascular Research Fund

**Disclosures:** *Yiannis S. Chatzizisis:* Speaker honoraria, advisory board fees, and research grant from Boston Scientific Inc.; Advisory board fees and research grant from Medtronic Inc.; Issued U.S. patent (No. 11,026,749) and international patent pending (application No. PCT/US2020/057304) for the invention entitled “Computational simulation platform for the planning of interventional procedures”; Co-founder of ComKardia Inc. All other authors have no relevant conflict of interest to disclose.

**Corresponding author:**

*Yiannis Chatzizisis, MD, PhD*

Division of Cardiovascular Medicine

University of Miami Health System

Leonard M. Miller School of Medicine

University of Miami

1120 NW 14^th^ Street, Suite 1124, Miami, FL, 33136

Tel: 305-243-2275

Email: [ychatzizisis@icloud.com](mailto:ychatzizisis@icloud.com)

**Figure S1. Original Lumen diameter of IVUS vs. μCT reconstructed silicone models.**

| 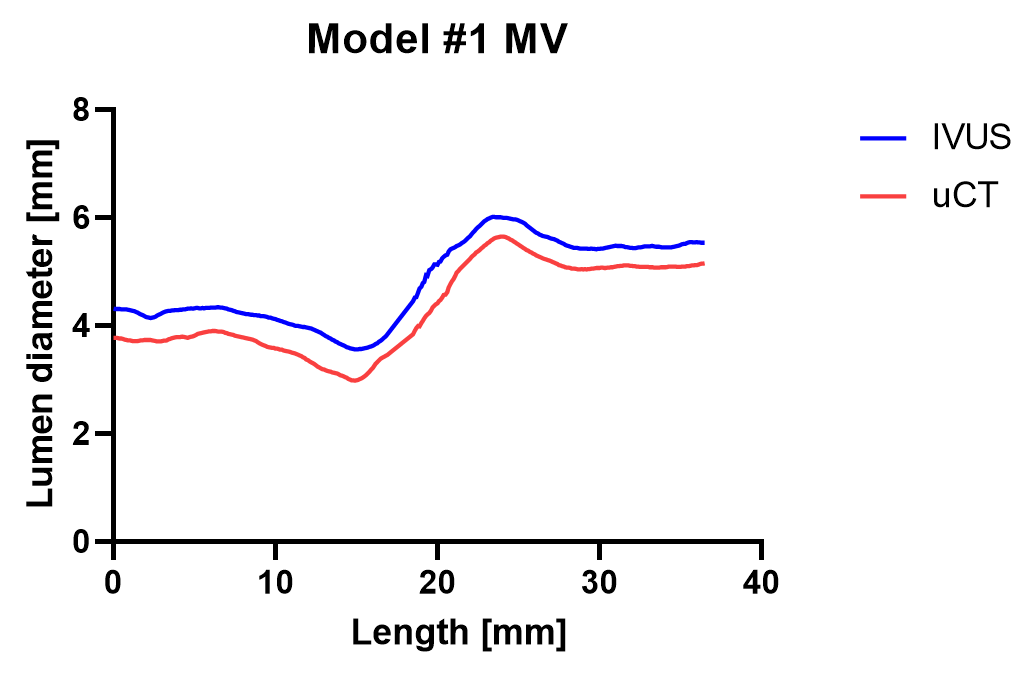 | 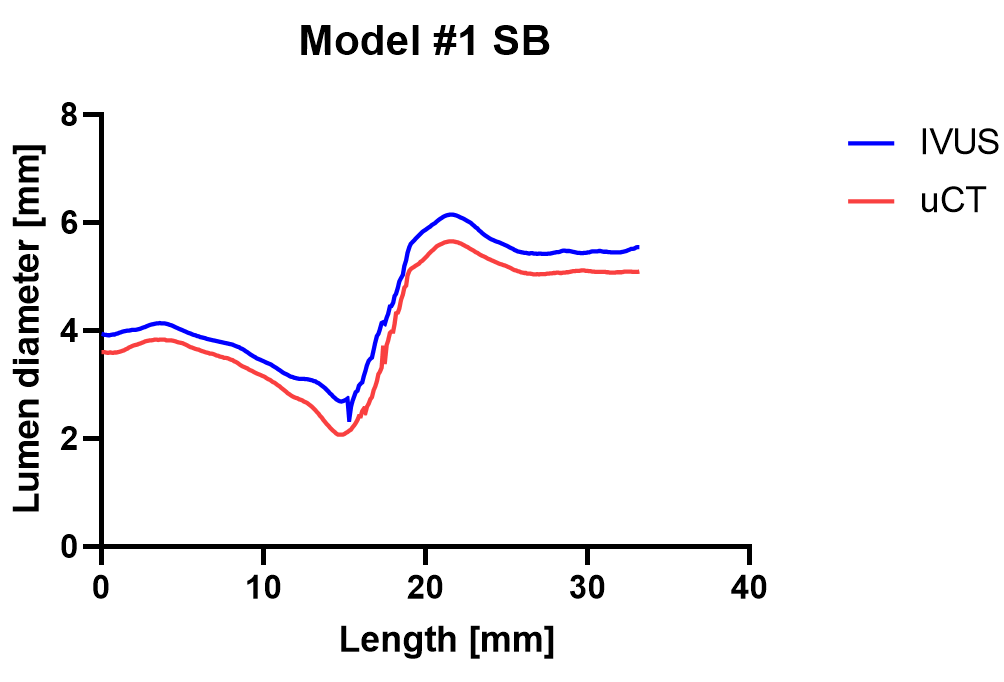 |
| --- | --- |
| 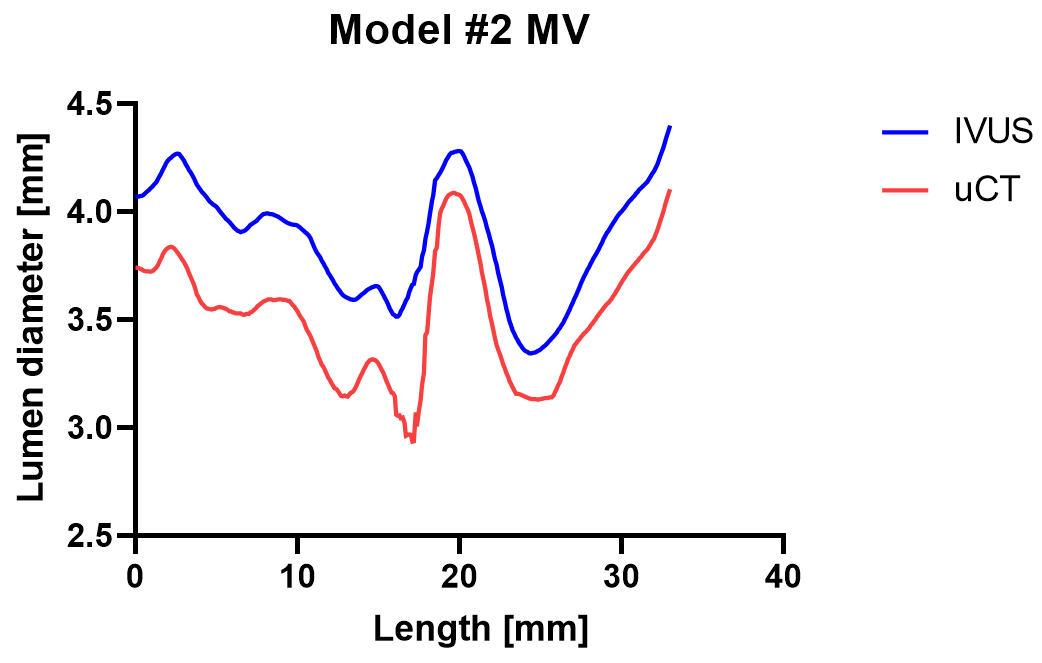 | 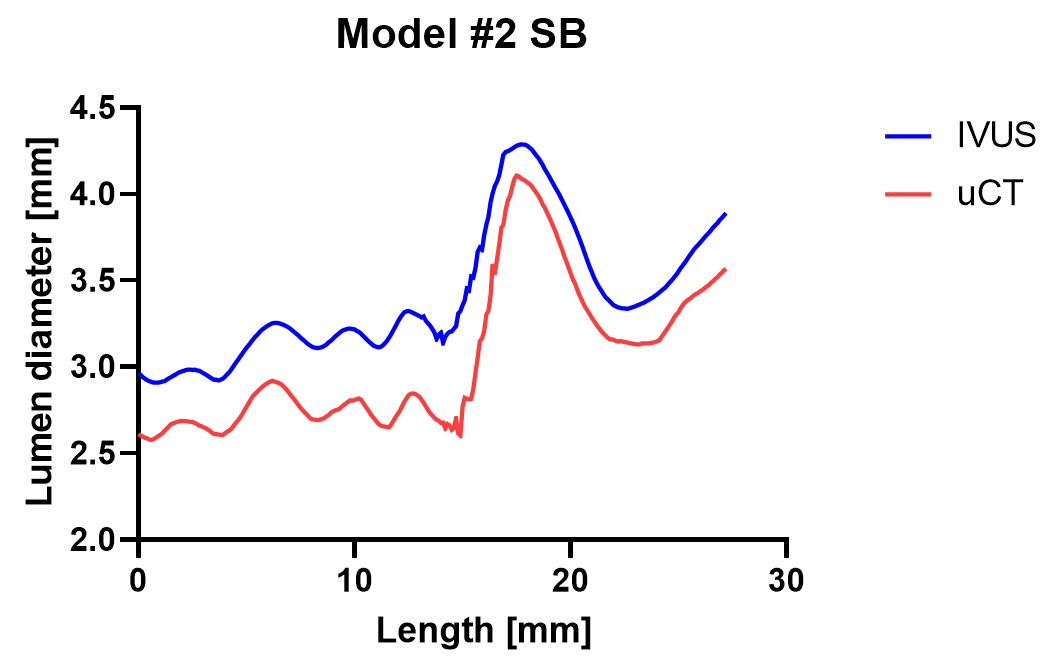 |
| 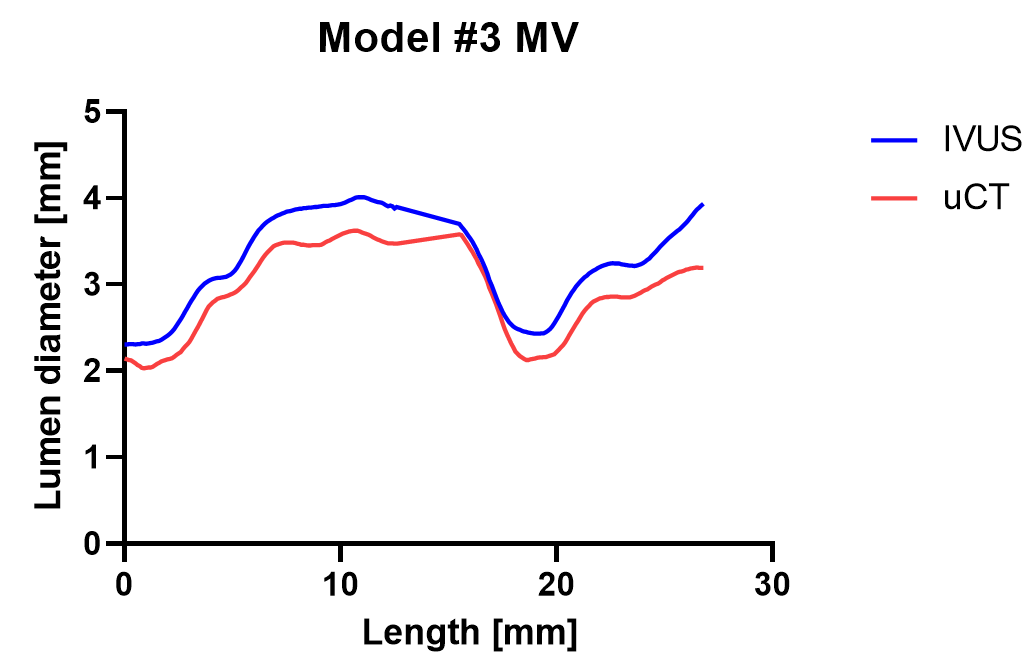 | 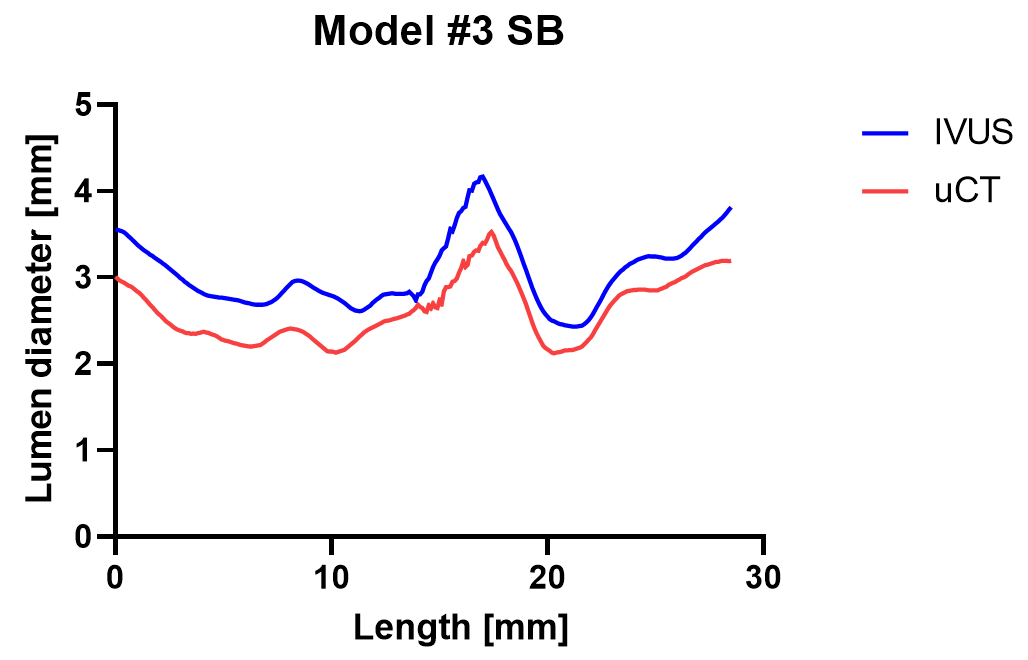 |

**Figure S2. Correction of the twisting effect of the IVUS frames in clinical cases due to the pulsatile motion of the coronary arteries.** (a and c) Before twisting correction. (b and d) After twisting correction.


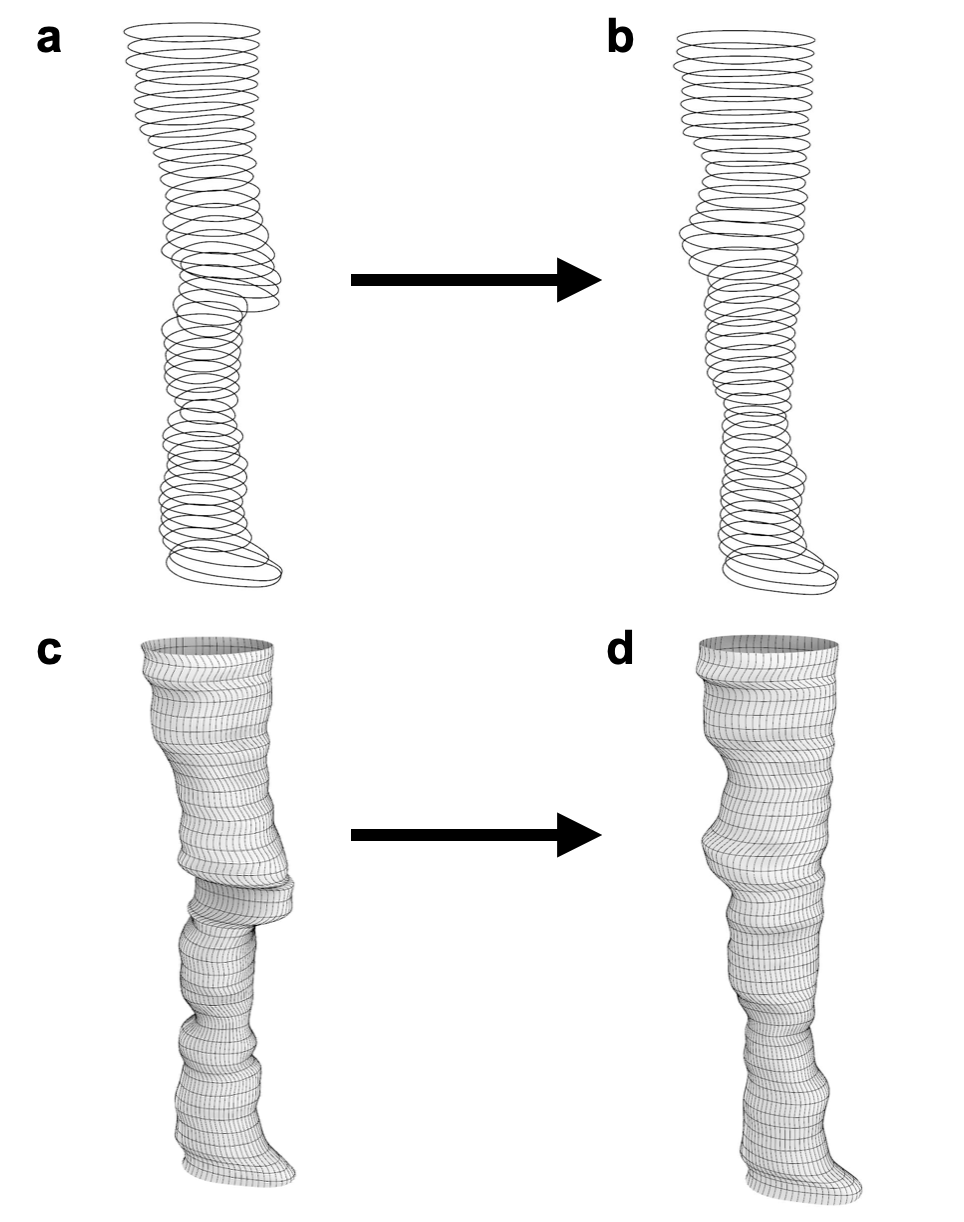
**Figure S3. Bland-Altman analysis of median, 25^th^ percentile, 75^th^ percentile shape ratio for in-vitro IVUS vs. μCT reconstructed models.**


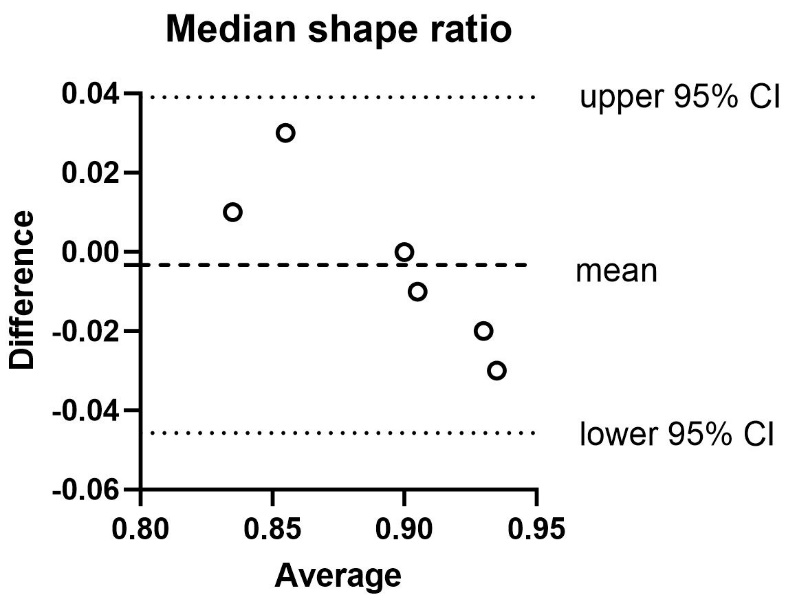

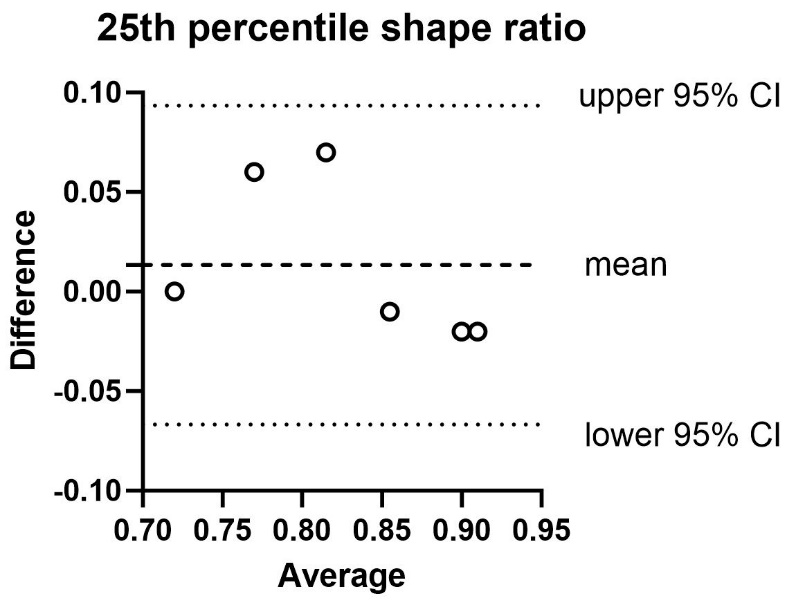

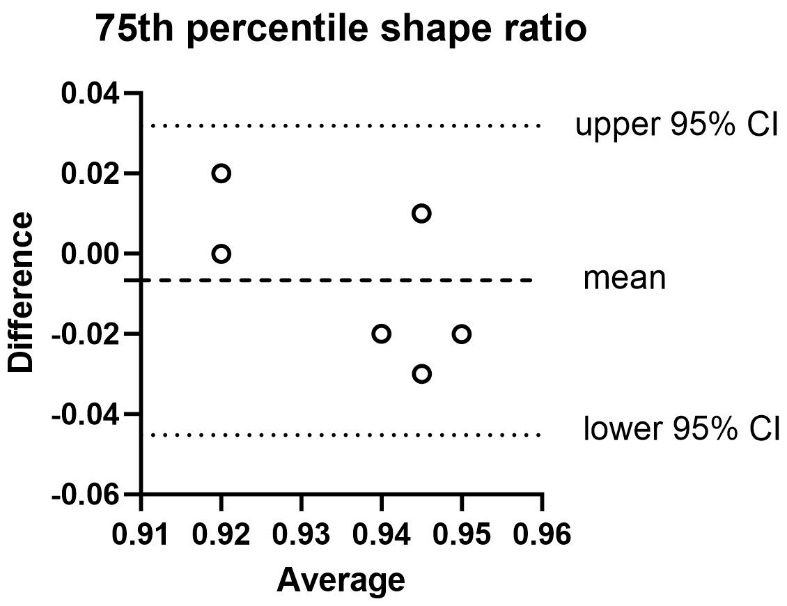


**Figure S4. An example of direct shape modification based on SubD**. (a) Mapping back checking of clinical case #2 lumen, which has been reconstructed and converted to SubD. (b) A section of the lumen is superimposed on the mapped back IVUS frame. (c) After a SubD facet is chosen, we can modify the local lumen shape directly by moving the facet with the reference of the IVUS frame. The SubD vertices and edges can also be chosen for shape modification.


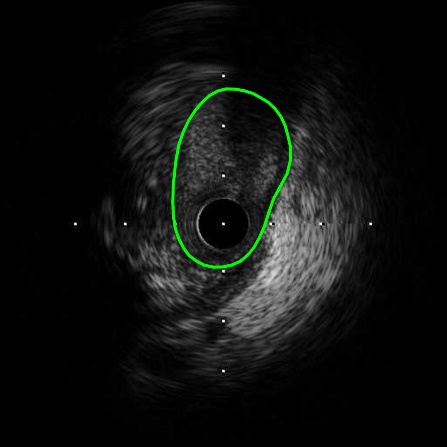

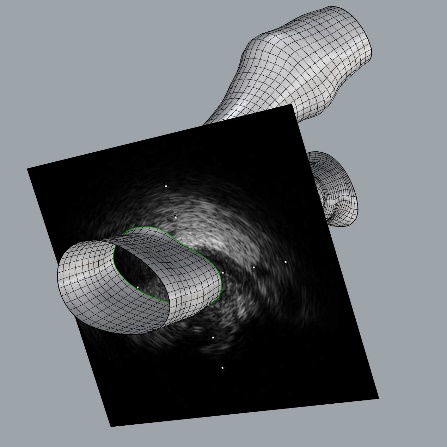

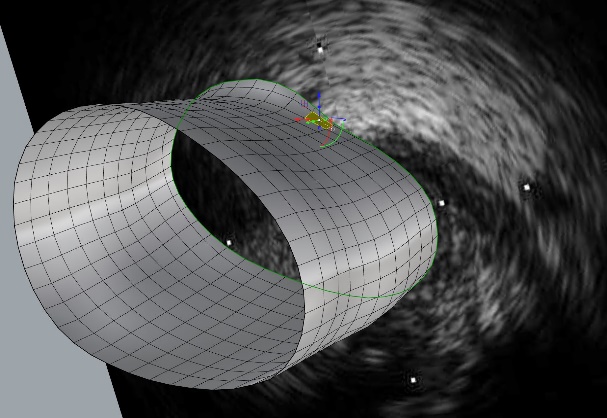


**(a)**

**(b)**

**(c)**

**Table S1. Patient-specific silicone and real patient coronary bifurcations in validation, reproducibility and feasibility studies.**

| **Vessel number** | **Coronary bifurcation** | |
| --- | --- | --- |
| **Silicone model** | Main vessel | Side branch |
| **#1** | Left anterior descending artery | First diagonal artery |
| **#2** | Left anterior descending artery | Second diagonal artery |
| **#3** | Left anterior descending artery | Third diagonal artery |
| **Clinical case** | Main vessel | Side branch |
| **#1** | Left anterior descending artery | Left circumflex artery |
| **#2** | Left anterior descending artery | Left circumflex artery |
| **#3** | Left anterior descending artery | Left circumflex artery |
